# Silver-Doped Diamond-Like Carbon Coatings on Ti_6_Al_4_V Alloy with Enhanced Antibacterial and Cytocompatible Properties

**DOI:** 10.1021/acsomega.5c10735

**Published:** 2026-03-19

**Authors:** Rossana Reim Del’ Gaudio Pignataro, Mariana Vegian, Diego Morais Silva, André Luis de Jesus Pereira, Argemiro Soares da Silva Sobrinho, Laura Soares Souto Lepesqueur, Cristiane Yumi Koga-Ito, Lafayette Nogueira Júnior

**Affiliations:** † Department of Dental Materials and Prosthodontics, Institute of Science and Technology, 28108 São Paulo State University (UNESP), São José dos Campos, SP 12245-000, Brazil; ‡ Department of Environmental Engineering, Institute of Science and Technology, São Paulo State University (UNESP), São José dos Campos, SP 12245-000, Brazil; § Plasmas and Processes Laboratory (LPP), Department of Physics, Aeronautics Institute of Technology (ITA), São José dos Campos, SP 12228-900, Brazil

## Abstract

To address persistent challenges in the longevity of biomedical and dental implants, particularly those related to bacterial colonization at metal interfaces, this study investigates the surface modification of Ti_6_Al_4_V alloy using diamond-like carbon (DLC) and silver-containing DLC (Ag-DLC) films deposited by plasma-enhanced chemical vapor deposition (PECVD) with a SiC_*x*_O*y* interlayer to promote adhesion. Raman spectroscopy and energy-dispersive X-ray spectroscopy (EDS) confirmed the formation of amorphous carbon coatings and verified the presence and spatial homogeneity of silver in the Ag-DLC films. Stylus profilometry and scratch testing indicated successful coating formation and favorable tribological performance, with Ag-DLC exhibiting a modest increase in friction coefficient relative to DLC. Contact-angle measurements revealed hydrophobic surfaces and a small decrease in surface energy following silver incorporation. Antibacterial assays against *Enterococcus faecalis* (ATCC 29212) demonstrated a significant reduction in viable colony-forming units on both DLC- and Ag-DLC-coated surfaces compared with uncoated Ti_6_Al_4_V (*p* < 0.05), while no statistically significant difference was observed between DLC and Ag-DLC under the tested conditions. SEM analysis showed denser bacterial adhesion on Ag-DLC surfaces, consistent with their measured topography; however, SEM and CFU data alone do not establish a definitive antibacterial killing mechanism. Cytocompatibility assays using NIH-3T3 fibroblasts over 24 h confirmed noncytotoxic behavior according to ISO 10993-5:2009. Overall, PECVD-deposited DLC and Ag-DLC coatings on Ti_6_Al_4_V combine favorable tribological behavior with reduced bacterial viability and short-term cytocompatibility, supporting their further evaluation for titanium-based implant surface engineering.

## Graphical abstract

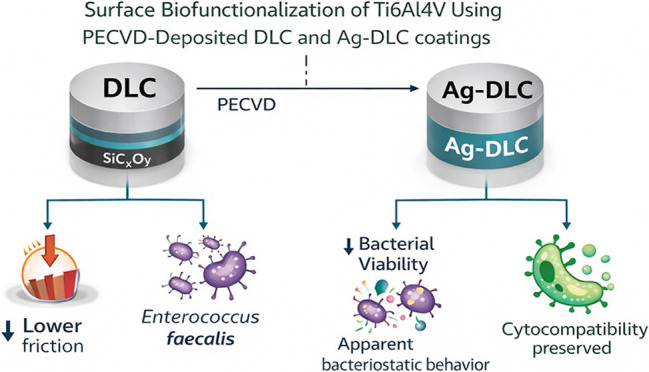

## Introduction

The long-term success of implant-supported rehabilitations relies on the mechanical and biological stability of the implant-abutment system. Micromovements and microgaps at this junction favor bacterial infiltration and persistent biofilm formation, processes that are closely associated with peri-implant inflammation and marginal bone loss. Surface modification strategies have therefore been extensively investigated to minimize microbial colonization without compromising biocompatibility, particularly through nanostructured coatings capable of modifying surface energy and hindering bacterial adhesion.^1–4^ Bacterial leakage at the implant-abutment interface remains a major clinical challenge, creating a microenvironment conducive to the formation of complex and resistant biofilms. Among microorganisms implicated in peri-implant infections, *Enterococcus faecalis* is notable for its ability to adhere to metallic and ceramic surfaces and for its resistance to pH variations, nutrient deprivation, and multiple antimicrobial agents.^5,6^ The dense extracellular matrix of *E. faecalis* biofilms limits antimicrobial penetration and enables bacterial persistence even after conventional disinfection, contributing to chronic inflammation and peri-implant bone loss.^2–7^ Accordingly, the development of surface coatings that reduce bacterial viability while maintaining cytocompatibility is essential for improving the biological stability of implant-supported systems.^8^ Diamond-like carbon (DLC) coatings have gained prominence as surface modifications for titanium and its alloys due to their exceptional tribological and biological characteristics. DLC exhibits high hardness, low friction coefficient, corrosion resistance, and chemical inertness, while maintaining excellent biocompatibility.^9,10^ These intrinsic material properties result in stable, often hydrophobic surfaces that can mitigate micromovements and deter bacterial attachment. However, achieving robust and stable adhesion between DLC coatings and metallic substrates remains challenging due to thermal and chemical incompatibilities, thereby motivating the development of interlayers and dopants to enhance coating performance.^11,12,18^

The incorporation of noble metals such as silver (Ag) into DLC matrices represents a promising strategy to introduce antimicrobial functionality^28^ while preserving the advantageous mechanical properties of carbon-based coatings. In many systems, antibacterial effects are attributed to silver present at or near the surface and, when applicable, to the release of silver species.^13–15^ Importantly, the magnitude and kinetics of silver release depend strongly on film architecture, silver content, and testing environment, and therefore must be experimentally verified for each coating system to ensure antimicrobial efficacy without inducing cytotoxicity.^16,17^

Accordingly, this study aimed to comprehensively investigate the physicochemical, antimicrobial, and cytocompatible properties of DLC and Ag-DLC thin films deposited on Ti_6_Al_4_V alloy discs using plasma-enhanced chemical vapor deposition (PECVD). Specifically, the objectives were to (i) deposit DLC and Ag-DLC coatings using a SiC_*x*_O*y* interlayer; (ii) characterize coating structure, composition, surface wettability and energy, and tribological behavior; (iii) evaluate antibacterial performance against *E. faecalis* using colony-forming unit (CFU) quantification and SEM analysis; and (iv) assess short-term in vitro cytocompatibility using NIH-3T3 fibroblasts according to the ISO 10993-5:2009 criterion.

## Experimental Section

This study was conducted at the Institute of Science and Technology, São Paulo State University (UNESP), School of Dentistry, São José dos Campos, Brazil. Two types of diamond-like carbon (DLC) films, pure DLC and silver-doped DLC (Ag-DLC), were deposited on Ti_6_Al_4_V alloy discs using plasma-enhanced chemical vapor deposition (PECVD). The procedures followed standardized protocols to ensure reproducibility and controlled film characteristics.

Sample Preparation. Ti_6_Al_4_V discs (19 mm diameter × 1 mm thickness) were machined from cylindrical bars, sequentially ground with silicon carbide papers of 600, 1200, and 2000 grit, and polished using universal polishing paste (Ivoclar Vivadent, Liechtenstein) to a mirror finish. Samples were ultrasonically cleaned in ethanol for 15 min and air-dried. A thin silicon-based interlayer (SiC_*x*_O*y*) was deposited prior to DLC or Ag-DLC coating to enhance adhesion between the metallic substrate and the carbon-based films.

Film Deposition. The PECVD process was performed at the Plasmas and Processes Laboratory (LPP), Technological Institute of Aeronautics (ITA), São José dos Campos, Brazil. The custom-built PECVD reactor was equipped with two power supplies: a pulsed DC (25 kHz, Advanced Energy Pinnacle Plus 5 kW × 5 kW, USA) for plasma generation and a DC supply (FFCT 1000-100i, Brazil) to activate a hollow silver cathode for Ag incorporation (Figure 1). Sample temperature was monitored by a type-K thermocouple, and gas flows were controlled by MKS flow controllers. The base pressure was maintained at 1.0 × 10^–3^ Torr, followed by heating to 530 °C and working pressure of 3 Torr. A SiC_*x*_O*y* interlayer (∼0.3 μm) was formed using tetramethylsilane (TMS) vapor for 10 min. DLC deposition was performed using Ar (60 sccm) and CH_4_ (3.8 sccm) at 0.6 Torr, pulsed DC 18 W for 20 min. Ag-DLC followed identical conditions, with the hollow cathode energized at ∼190 W DC for silver doping. After deposition, the chamber cooled to 80 °C under vacuum.

**Figure 1:**
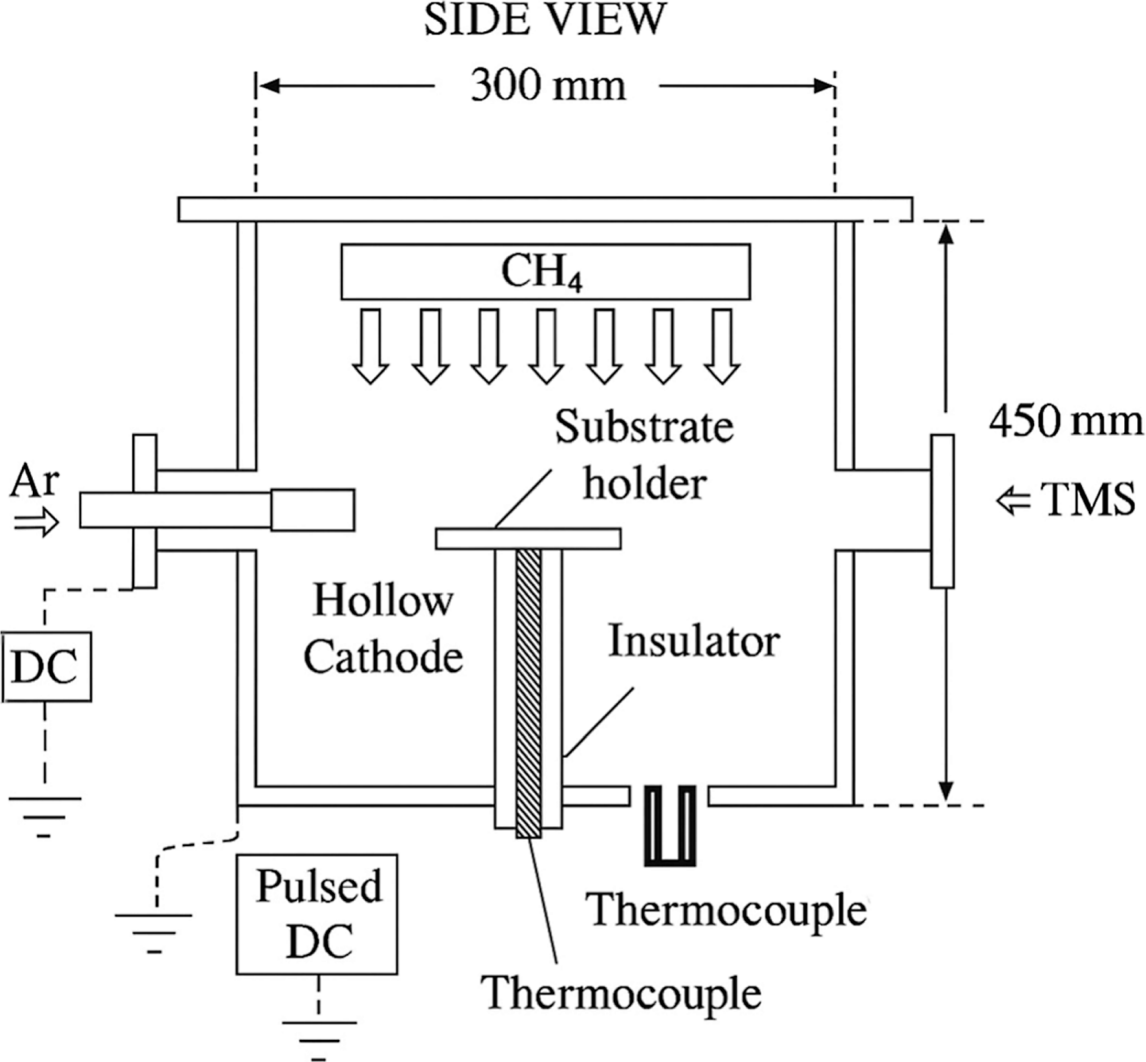
Schematic diagram of the PECVD reactor showing main components and precursor gas inlets.

The deposition parameters used for DLC and Ag-DLC films are summarized in Table 1.

**Table 1:** Parameters Used for the Deposition of DLC Films on Ti_6_Al_4_V Alloy

activities	argon flow (sccm)	flow HMDSO (Torr)	pulsed DC power (W)	power DC (W)	temperature (°C)	time (minutes)	pressure (Torr)
warming up	60		190		25–550	∼40	0.9–1.0
interface (SiC*x*O*y*)	60	0.02	60		550	15	0.6
cooling 1	60				550–175	∼60	0.3
deposition DLC	60		18		175	20	0.6
deposition Ag-DLC	60		18	190	175	20	0.6
cooling 2	60				175–30	∼210	0.3

### Film Characterization

Film thickness and roughness were measured by stylus profilometry (KLA Tencor P-7). Adhesion was assessed by scratch testing (CETR UMT-2 Tribometer, Bruker, USA) using a Rockwell-type diamond stylus under progressive loading from 0–40 N. Raman spectroscopy (Horiba Evolution, 532 nm laser) confirmed the amorphous carbon structure via D (∼1350 cm^–1^) and G (∼1580 cm^–1^) bands. Contact angles were measured using a Ramé-Hart Model 500 goniometer with water and diiodomethane, and surface energy was calculated using the Owens–Wendt method. Chemical composition and Ag incorporation were evaluated by energy-dispersive X-ray spectroscopy (EDS, Oxford Instruments on Tescan XMU, 15 kV).

Microbiological Assays. *E. faecalis* (ATCC 29212) was used as the test microorganism. Cultures were grown in brain heart infusion (BHI) broth at 37 °C for 24 h. Discs were sterilized under UV for 10 min, immersed in bacterial suspensions (1 × 10^6^ CFU·mL^–1^), and incubated at 37 °C for 90 min with shaking (120 rpm) for adhesion, followed by 48 h biofilm formation with medium exchange after 24 h. After rinsing, adherent cells were detached by ultrasonication (3 × 20 s, 10 s intervals), serially diluted, and plated on BHI agar for CFU quantification.

### Scanning Electron Microscopy

After biofilm formation, discs were fixed in 2.5% glutaraldehyde (24 h), rinsed with 0.1 M phosphate buffer, dehydrated in graded ethanol (30–100%), dried at 65 °C, sputter-coated with gold (12 nm, Quorum SC7620), and imaged under a FEI Inspect S50 SEM at 10 kV and 3000×–5000× magnification.

### Cytocompatibility Assay

The MTT assay was performed using NIH-3T3 fibroblast cells (ATCC CRL-1658) cultured in Dulbecco’s Modified Eagle Medium (DMEM) with 10% fetal bovine serum and 1% penicillin/streptomycin. Cells (2 × 10^5^/well) were seeded in 6-well plates and incubated with coated or uncoated discs for 24 h. After contact, MTT reagent (0.5 mg·mL^–1^) was added for 3 h, and formazan crystals were solubilized with DMSO. Absorbance was measured at 570 nm (Anthos 2020, Austria). Two independent experiments were performed in triplicate. Results were analyzed according to ISO 10993-5:2009 cytotoxicity criteria.

## Results

### Film Morphology and Thickness

Mechanical profilometry revealed that both diamond-like carbon (DLC) and silver-doped DLC (DLC-Ag) coatings exhibited an average thickness of 180 ± 20 nm (Figure 2). The roughness parameters Rp and Ra are summarized in Table 2. The DLC-Ag coating showed a higher Rp value (0.341 μm) compared with pure DLC (0.100 μm), whereas Ra values remained similar between groups. The more prominent peak intensity observed for DLC-Ag indicates the presence of higher surface peaks compared with pure DLC.

**Figure 2:**
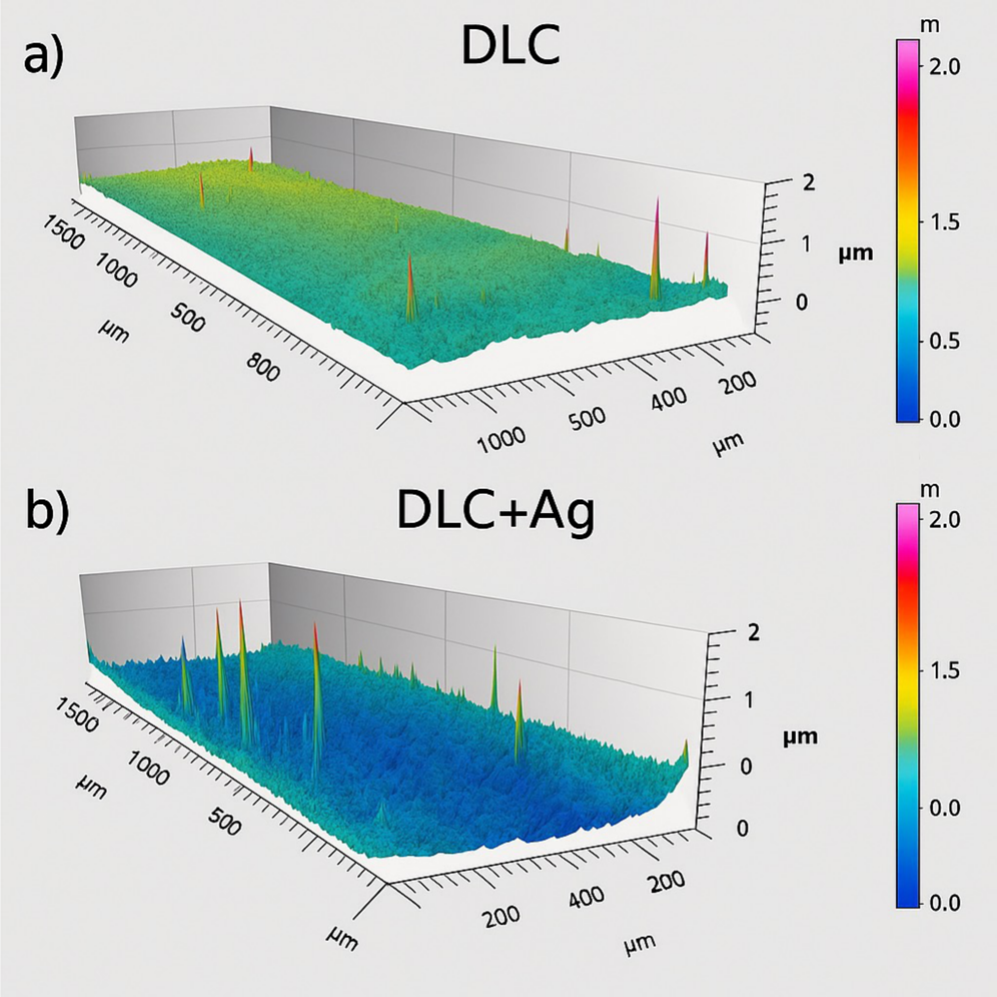
Surface morphology and roughness profiles of (a) DLC and (b) Ag-DLC films obtained by stylus profilometry.

**Table 2:** Surface Roughness Parameters of DLC and Ag-DLC Coatings

sample	Rp^a^ (μm)	Ra (μm)
DLC	0.100	0.0128
Ag-DLC	0.341	0.0138

a Rp represents the mean peak height within the scanned area (1500 μm × 500 μm).

### Tribological Behavior

As reported in the literature, DLC films generally present high hardness, low friction coefficients, and good wear resistance. The expected dynamic friction coefficient for Ti_6_Al_4_V is approximately 0.41,^29^ while the coatings analyzed here displayed significantly lower values: 0.12 ± 0.10 for DLC and 0.154 ± 0.008 for DLC-Ag (Figure 3). These results indicate strong adhesion and favorable tribological performance for both coatings, with the marginally higher friction observed for Ag-doped films likely reflecting subtle differences in surface chemistry and energy distribution.

**Figure 3:**
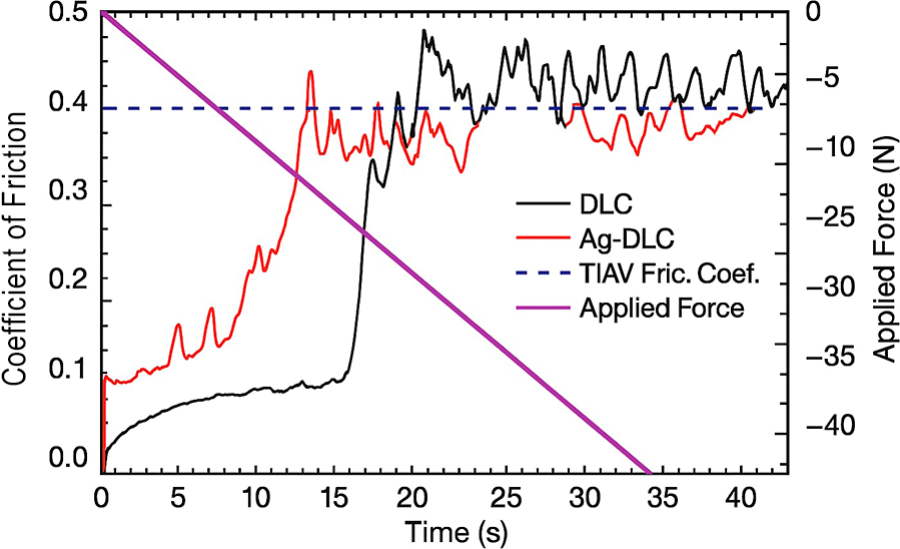
Friction coefficient and applied load during scratch test; coating delamination is observed when the friction coefficient reaches the dynamic value for Ti_6_Al_4_V (∼0.41).

### Raman Spectroscopy

Raman spectra (Figure 4, Table 3) exhibited the characteristic D and G bands of amorphous carbon films at approximately 1360 cm^–1^ and 1580 cm^–1^, respectively. The G peak corresponds to E2g stretching vibrations of sp^2^ carbon bonds, while the D peak arises from disordered A1g breathing modes of sp^2^ carbon rings. In the DLC-Ag film, two additional components at 1215 cm^–1^ and 1476 cm^–1^ were required for adequate fitting, attributed to C=C stretching and C–H wagging vibrations, respectively. The narrower fwhm and upshifted G band observed in DLC-Ag are consistent with changes in graphitic ordering reported for metal-doped DLC films.

**Figure 4:**
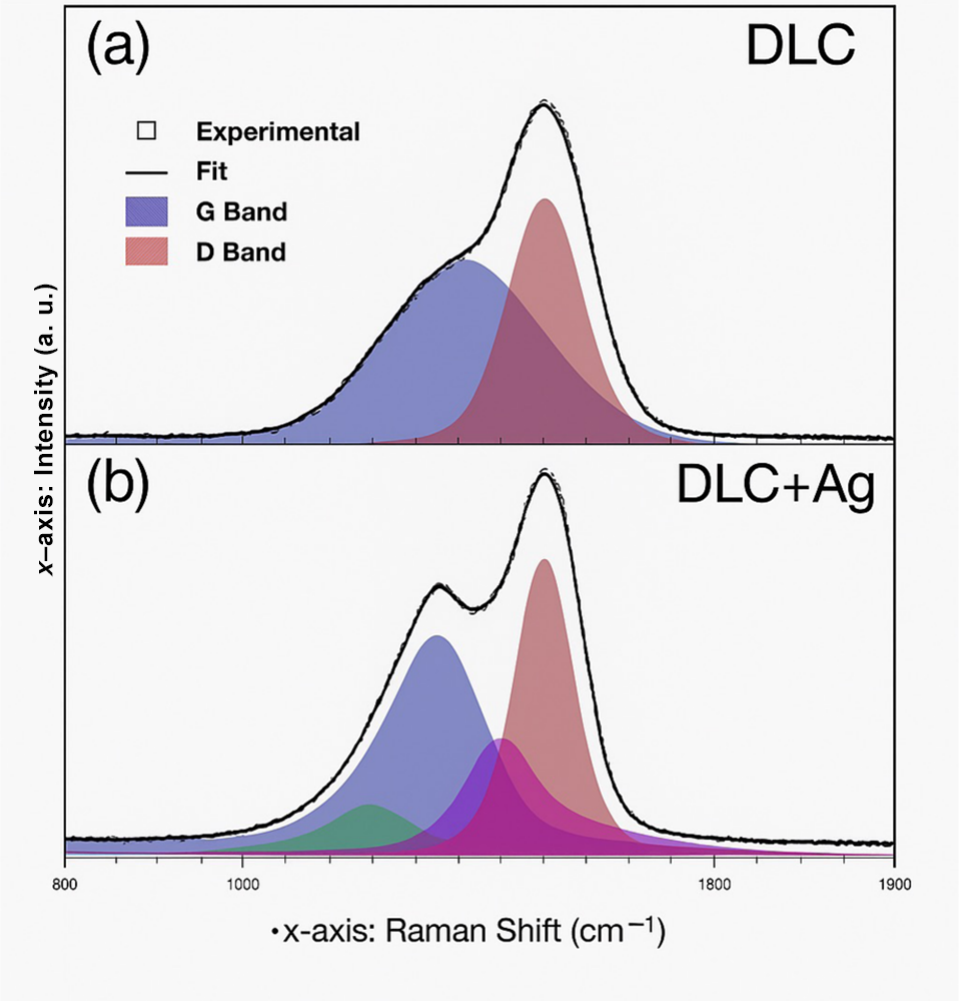
Raman spectra of (a) DLC and (b) Ag-DLC coatings excited with a 532 nm green laser. Deconvolution of D and G bands performed using Voigt functions.

**Table 3:** Results of the Fitting of the G and D Bands Performed in the Raman Spectroscopy Measurements of DLC and Ag-DLC Coatings

sample	position peak G (cm^–1^)	fwhm^a^ (cm^–1^)	position peak D (cm^–1^)	fwhm (cm^–1^)	AD/AG^b^	ID/IG^c^
DLC	1548.6 ± 0.2	156 ± 1	1374 ± 2	346 ± 3	1.6 ± 0.5	0.75
Ag-DLC	1574.5 ± 0.7	119 ± 1	1356 ± 3	226 ± 18	2.0 ± 0.5	0.73

a Full width at half maximum.

b Ratio between areas.

c Ratio between intensities.

### Contact Angle and Surface Energy

Contact angle measurements (Figure 5) showed that DLC-Ag surfaces were more hydrophobic than pure DLC. Using deionized water and diiodomethane as probe liquids, surface energy values were calculated via the Owens–Wendt method, revealing an approximate decrease of 2 mN·m^–1^ after silver doping. This reduction reflects a decrease in the polar component of surface energy and lower wettability of the Ag-DLC surface.

**Figure 5:**
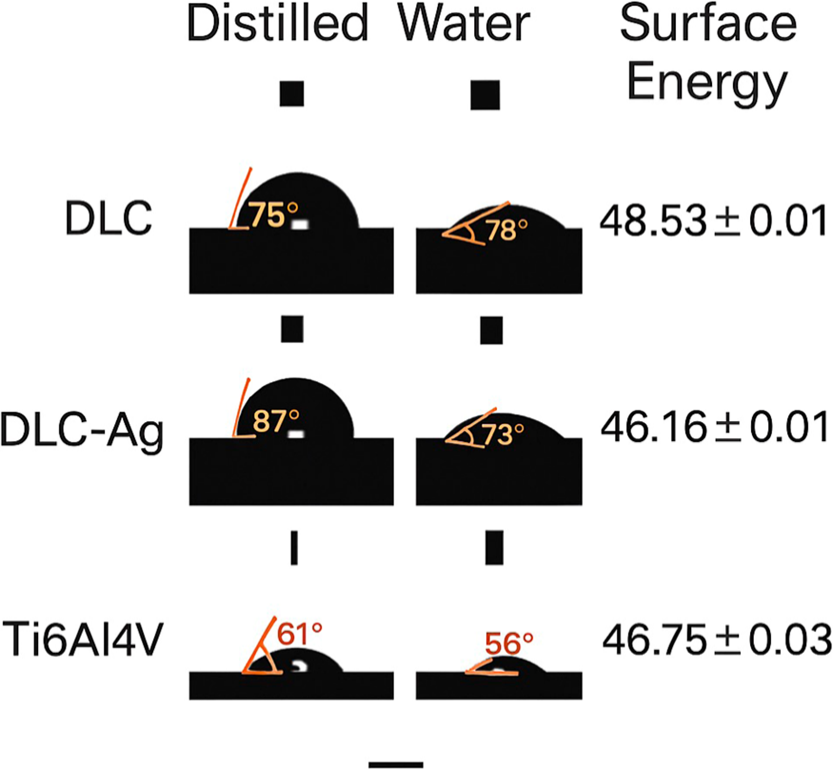
Water and diiodomethane contact angle measurements showing surface wettability of DLC and Ag-DLC coatings.

### Elemental Composition (EDS)

Energy-dispersive X-ray spectroscopy (EDS) confirmed uniform silver incorporation into the carbon matrix (Figure 6). Elemental mapping demonstrated homogeneous distribution of carbon, silicon, and silver across the coating, indicating successful and stable silver doping throughout the film.

**Figure 6:**
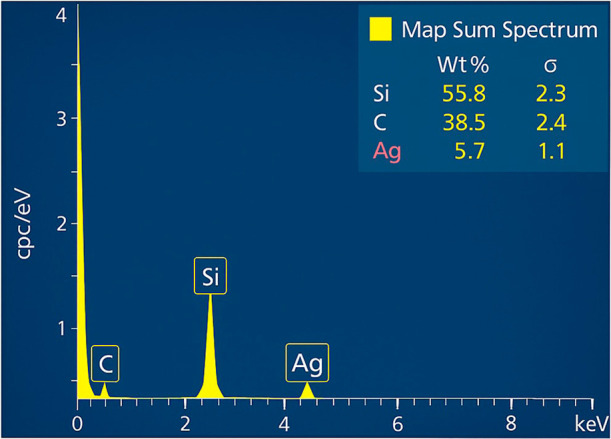
Quantitative EDS spectra confirming silver incorporation into the carbon matrix.

### Antibacterial Activity

Bacterial plating on BHI agar after ultrasonic washing of the disc surfaces resulted in typical *E. faecalis* colony growth. Quantification of colony-forming units (CFU·mL^–1^) showed a significant reduction (*p* < 0.05) for both DLC and DLC-Ag coatings compared with the uncoated control (Figure 7). No statistically significant difference was observed between the two coated groups under the tested conditions.

**Figure 7:**
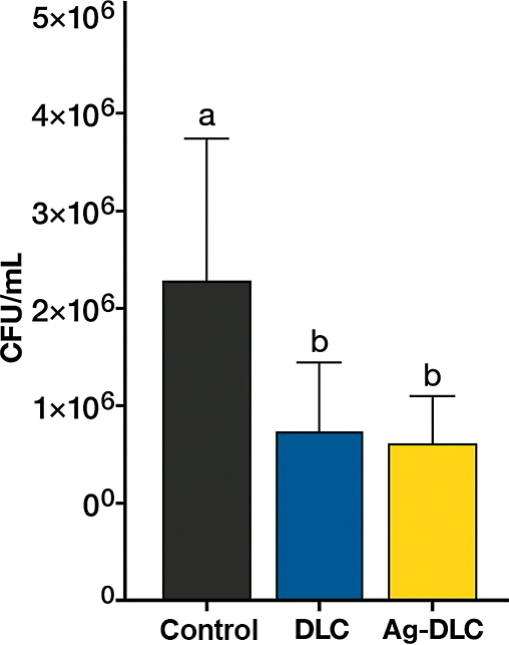
CFU counts of *Enterococcus faecalis* after 48 h incubation on uncoated, DLC, and Ag-DLC surfaces (*p* < 0.05).

### SEM Analysis

SEM micrographs (Figure 8) revealed bacterial adhesion and biofilm formation on all surfaces. The DLC-Ag coating exhibited a denser bacterial layer compared with the DLC and control groups. These observations are consistent with the CFU analysis and suggest an antibacterial effect associated with the coatings (Figure 8).

**Figure 8:**
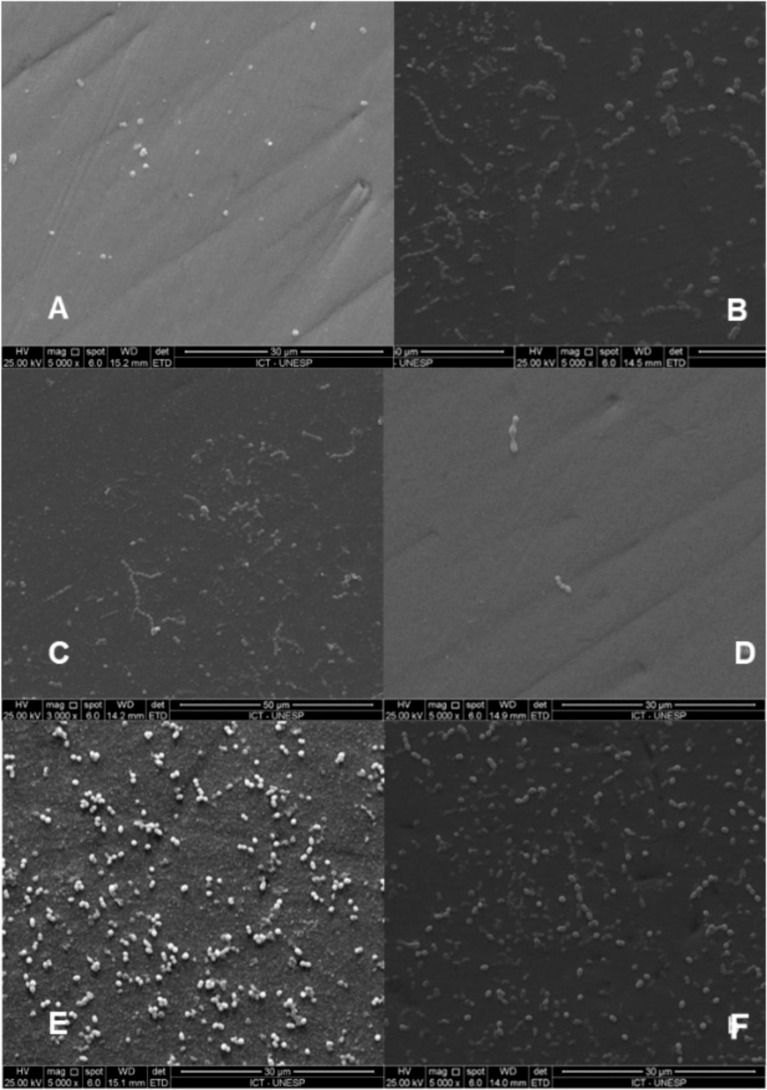
SEM micrographs of (A,B) control, (C,D) DLC, and (E,F) Ag-DLC discs showing biofilm morphology.

### Cytotoxicity

The MTT assay (Figure 9) demonstrated that none of the coatings exhibited cytotoxic behavior after 24 h of contact with NIH-3T3 fibroblasts. The mean cell viability was 142% for the uncoated control, 116% for DLC, and 71.5% for DLC-Ag. Although Ag-DLC showed a moderate reduction in cell viability, all values remained above the 70% threshold defined by ISO 10993-5:2009 for noncytotoxic materials.

**Figure 9:**
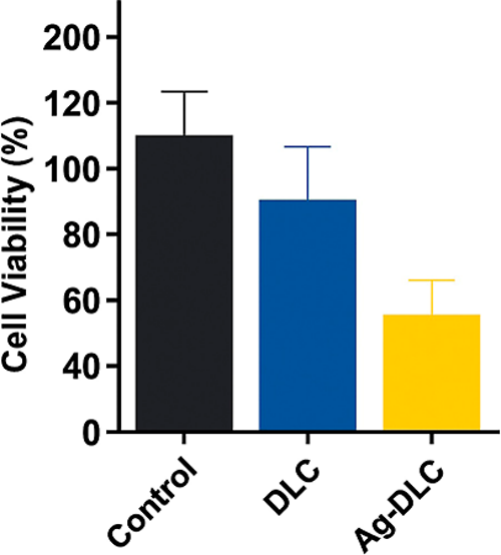
NIH-3T3 fibroblast viability after 24 h contact with uncoated Ti_6_Al_4_V, DLC, and Ag-DLC samples according to ISO 10993-5:2009.

## Discussion

The structural, tribological, and biological behavior of the DLC and Ag-DLC coatings deposited on Ti_6_Al_4_V alloy reflects the multifunctional role of diamond-like carbon films in biomedical surface engineering.^22^ Raman spectroscopy confirmed the amorphous carbon structure of both coatings through the characteristic D and G bands, corresponding to sp^2^ and sp^3^ carbon bonds, corroborating the formation of the DLC phase as widely reported for hydrogenated and metal-doped coatings deposited by PECVD.^20,21,23,24^ Subtle variations in band position and width observed for Ag-DLC suggest localized modifications in carbon bonding configuration associated with silver incorporation, in agreement with earlier studies on metal-doped DLC films, although stress relaxation effects were not directly quantified in the present work.^16,17,41^

Tribological testing demonstrated a pronounced reduction in friction coefficient for both DLC and Ag-DLC coatings compared with uncoated Ti_6_Al_4_V, corroborating the well-established lubricious behavior of DLC surfaces.^29–31^ The slightly higher friction coefficient observed for Ag-DLC is consistent with literature reports indicating that metal incorporation may modify the carbon network and interfacial shear behavior.^12,16^ Importantly, despite this increase, friction values remained substantially lower than those of the bare alloy, confirming the suitability of both coatings for applications involving repetitive mechanical loading.^30,31^

Surface wettability and surface energy analyses revealed that silver incorporation slightly increased hydrophobicity^24^ and reduced the polar component of surface energy,^26^ a trend previously reported for metal-doped DLC coatings and known to influence bacterial adhesion behavior.^10–13,19,22,25^ Stylus profilometry indicated the presence of localized surface peaks (Rp) that, in some instances, approached or exceeded the nominal coating thickness. These features are interpreted as isolated surface asperities rather than representative nanoscale roughness and may be associated with substrate-related particulates present prior to deposition, subsequently conformally coated during the PECVD process. As stylus profilometry has limited resolution at the nanoscale, complementary techniques such as atomic force microscopy would be required to more accurately resolve surface roughness distributions.^32–34,38,39^

From a biological perspective, both DLC and Ag-DLC coatings significantly reduced *E. faecalis* viability compared with uncoated Ti_6_Al_4_V, in line with previous studies demonstrating the antibacterial potential of carbon-based coatings.^3,5,7,12,24–28^ No statistically significant difference was observed between DLC and Ag-DLC in CFU counts under the conditions tested. SEM analysis revealed increased bacterial adhesion on Ag-DLC surfaces, likely associated with the combined effects of surface roughness and hydrophobicity; however, this increased adhesion did not translate into higher bacterial viability.

This apparent discrepancy suggests that while Ag-DLC surfaces may permit bacterial attachment, bacterial proliferation is inhibited after adhesion. Importantly, the available data do not allow direct inference of antibacterial mechanisms. In the absence of Ag^+^ release measurements or fluorescence-based viability assays, the antibacterial behavior of Ag-DLC should be interpreted as indicative of an apparent bacteriostatic effect rather than a confirmed bactericidal mechanism.^24^ Nevertheless, the antimicrobial efficiency of silver ions and silver-based nanostructures has been widely demonstrated against different bacterial species, including *E. faecalis*, either alone or in combination with other agents.^40–42^ Accordingly, silver-related effects observed in the present study are discussed as being consistent with literature-reported antimicrobial activity rather than as direct evidence of ion-mediated killing.^36,37^

EDS analysis confirmed the homogeneous incorporation of silver into the carbon matrix, with a uniform distribution of carbon, silicon, and silver elements across the analyzed surface. The absence of metallic agglomerates indicates a stable and controlled deposition process.^18^ This uniform dispersion is crucial, as heterogeneous silver distribution can lead to localized stress concentrations, compromise mechanical integrity, and result in an uncontrolled burst release of Ag^+^ ions, potentially causing cytotoxicity or rapid depletion of the antimicrobial reservoir.^14,17^

Regarding the overall antibacterial performance, Velioglu et al.^35^ proposed two complementary mechanisms for the antibacterial effect of DLC coatings: (i) An extremely smooth surface limits bacterial adhesion and biofilm development, and (ii) the coating acts as a physical barrier, isolating the Ti_6_Al_4_V alloy, which is inherently more prone to bacterial colonization. Our findings, particularly the significant reduction in microbial proliferation on both coated samples regardless of subtle microtexture differences, are consistent with these mechanisms.

Cytocompatibility assessment using NIH-3T3 fibroblasts demonstrated that both coatings maintained cell viability above the ISO 10993–5 threshold for noncytotoxic materials after 24 h of exposure. Although Ag-DLC exhibited reduced viability compared with pure DLC, values remained within acceptable limits. These results are consistent with prior reports indicating that silver-doped DLC films can preserve cytocompatibility when silver incorporation is limited.^11,14,15,23,24^ Nevertheless, longer-term studies and evaluation using osteoblast-lineage cells are necessary to assess the relevance of these coatings for osseointegration.

### Study Limitations

This study presents several limitations that should be considered when interpreting the results. First, silver ion release kinetics were not quantified, precluding a direct correlation between Ag incorporation and antimicrobial mechanisms. Second, surface roughness was assessed by stylus profilometry, which has limited resolution for nanoscale features; atomic force microscopy would provide more detailed topographical information. Third, antibacterial activity was evaluated using CFU counts and SEM imaging, which do not directly assess bacterial viability at the single-cell level; fluorescence-based live/dead assays could provide complementary insights. Finally, cytocompatibility was assessed after short-term exposure using fibroblast cells; longer-term studies and evaluation with osteoblast-lineage cells are warranted to further elucidate the biological performance of these coatings.

## Conclusion

Within the limitations of this in vitro investigation, we conclude that both diamond-like carbon (DLC) and silver-doped DLC (Ag-DLC) coatings were successfully deposited on Ti_6_Al_4_V alloy, achieving stable adhesion effectively promoted by the crucial SiC_*x*_O_*y*_ silicon interlayer. While silver incorporation slightly modified the surface topography and mechanical response, including a subtle increase in roughness and friction, it critically did not compromise film homogeneity or the characteristic Raman signatures of the DLC phase.

Our key findings demonstrate the dual functionality of these coatings: Both DLC and Ag-DLC significantly reduced *E. faecalis* colonization and biofilm formation compared to the uncoated control, exhibiting potent antibacterial behavior. Notably, despite Ag-DLC exhibiting increased bacterial adhesion, the observed strong reduction in viable colony-forming units underscores a predominant bacteriostatic mechanism of action for the silver-doped coatings. Furthermore, cytotoxicity results unequivocally confirmed the biocompatibility of both coatings in accordance with ISO 10993-5:2009 standards.

Therefore, these PECVD-deposited DLC and Ag-DLC coatings represent highly promising, multifunctional surface-engineering strategies for titanium-based biomedical implants. They offer a unique combination of enhanced resistance to bacterial colonization, maintained favorable mechanical properties, and verified biological performance, thus addressing critical challenges for the long-term durability and success of implantable devices.
